# In-depth analysis of swim bladder-associated microbiota in rainbow trout (*Oncorhynchus mykiss*)

**DOI:** 10.1038/s41598-019-45451-1

**Published:** 2019-06-20

**Authors:** Alejandro Villasante, Carolina Ramírez, Héctor Rodríguez, Natalia Catalán, Osmán Díaz, Rodrigo Rojas, Rafael Opazo, Jaime Romero

**Affiliations:** 1 Laboratorio de Biotecnología de Alimentos, Unidad de Alimentos, Instituto de Nutrición y Tecnología de los Alimentos (INTA), Universidad de Chile, Santiago, Chile; 2 Facultad de Medicina, Universidad de Chile, Programa de Anatomía y Biología del Desarrollo, Santiago, Chile; 3 Laboratorio de Patobiología Acuática, Departamento de Acuicultura, Facultad de Ciencias del Mar, Universidad Católica del Norte, Coquimbo, Chile

**Keywords:** Microbiome, Symbiosis

## Abstract

Our knowledge regarding microbiota associated with the swim bladder of physostomous, fish with the swim bladder connected to the esophagus via the pneumatic duct, remains largely unknown. The goal of this study was to conduct the first in-depth characterization of the swim bladder-associated microbiota using high-throughput sequencing of the V4 region of the 16 S rRNA gene in rainbow trout (*Oncorhynchus mykiss*). We observed major differences in bacterial communities composition between swim bladder-associated microbiota and distal intestine digesta microbiota in fish. Whilst bacteria genera, such as *Cohnella*, *Lactococcus and Mycoplasma* were more abundant in swim bladder-associated microbiota, *Citrobacter*, *Rhodobacter* and *Clavibacter* were more abundant in distal intestine digesta microbiota. The presumptive metabolic function analysis (PICRUSt) revealed several metabolic pathways to be more abundant in the swim bladder-associated microbiota, including metabolism of carbohydrates, nucleotides and lipoic acid as well as oxidative phosphorylation, cell growth, translation, replication and repair. Distal intestine digesta microbiota showed greater abundance of nitrogen metabolism, amino acid metabolism, biosynthesis of unsaturated fatty acids and bacterial secretion system. We demonstrated swim bladder harbors a unique microbiota, which composition and metabolic function differ from microbiota associated with the gut in fish.

## Introduction

In teleost species, the swim bladder is a unique gas-filled organ crucial for regulation of buoyancy, equilibrium and position of fish in the water column by modulating whole-body density^1,2^. The swim bladder emerges as an evagination of the digestive tract during early larvae development^2^. Previous studies conducted in the zebrafish model, have described the swim bladder as homolog structure of the tetrapod lung^1,3^. The swim bladder exhibits similar developmental patterns to early mammalian lung development. Indeed, both organs arise from single or paired pouch of the cranial part of the digestive tube during embryonic development^1,3^. In teleost fish, the pneumatic duct connects the esophagus to the swim bladder chamber, and emerges at the end of the embryonic stage^3^. The ontogenic timing of the pneumatic duct differs between physoclists and physostomous species. In physoclists, the pneumatic duct disappears after the first inflation of the swim bladder during larvae stage, and thus the swim bladder remains closed and isolated from the digestive tract during adult stage^2^. In these species, the rete mirabile, a highly vascularized capillary network, constitutes the main mechanism to incorporate gases into the swim bladder^2^. On the other hand, in physostomous fish, the pneumatic duct remains connected to the esophagus during the entire life of fish^2,4,5^, and thus it constitutes a route for direct gas exchange between the environment and the swim bladder. Further, in physostomous, it has been described the major mechanism to inflate the swim bladder is through the mouth by “gulping” air from the water surface^2,6,7^. Therefore, environmental microbes, particularly from air, may get access into the swim bladder via the esophagus-pneumatic duct route in physostomous fish.

Most research conducted in fish microbiota has been focused on gut-associated microbiota and its role regulating physiology and health in host, particularly in aquaculture fish species^8^. These studies have demonstrated the intestinal microbiota diversity is complex and highly modulated by dietary ingredients^8^. However, our knowledge regarding microbiota associated with internal mucosa other than the gastro enteric tract, such as the swim bladder mucosa, remains largely unknown. Recently, we reported bacteria genera *Arthrobacter* and *Cellulosimicrobium* were observed in the swim bladder of the rainbow trout (*Oncorhynchus mykiss*)^9^. However, in that study, microbiota was identified using temporal temperature gradient gel electrophoresis (PCR-TTGE). PCR-TTGE has some limitations profiling bacterial communities in high resolution, and thus high-throughput sequencing technology for massive sequencing is preferred.

The main goal of this study was to identify major bacterial genera associated with the swim bladder in the rainbow trout using next generation sequencing technology. Second objective was to carry out a predictive metabolic pathways analysis (PICRUSt) of the microbiota associated with the swim bladder in rainbow trout.

## Material and Methods

### Fish and rearing conditions

The study was conducted in the recirculation freshwater system at the Instituto de Nutrición y Tecnología de los Alimentos (INTA) of Universidad de Chile. The fish management protocol was in accordance with the recommendations of Guide for the Care and Use of Laboratory Animals of the National Institutes of Health, and the Committee on the Ethics of Animal Experiments of Faculty of Agronomic Science, Universidad de Chile, approved the experimental setting. A total of 40 fish (226 ± 8.7 g), *Oncorhynchus mykiss*, were obtained from Piscícola Huililco Ltda. (Pucón, Region de la Araucanía, Chile), and evenly stoked in two 150-L fiberglass tanks (20 fish per tank). The tanks were supplied with well-aerated freshwater at a constant temperature (15 ± 0.5 °C; 12 L min^−1^; >90% oxygen saturation). Fish were kept under a photoperiod of 12 h light/12 h dark and fed a commercial diet during both the two-week acclimatization period and the four-week trial.

### Diets and feeding

A commercial, extruded diet and fulfilling the National Research Council nutritional requirements for rainbow trout^10^ was fed to duplicate tanks with 20 fish each during four weeks. The ingredients composition in diet was fishmeal (37%), soy protein concentrate (38%), wheat gluten (7.5%) and fish oil (14%). The diet proximate composition was crude protein 53.7%, lipid 17.7%, ash 10.2%, crude fiber 2.1%, and water 7.2%. The estimated gross energy was 21 MJ/kg. Fish were fed by hand three times per day to apparent satiation, six day per week during the trial.

### Sampling procedure

After 18 hours of fasting, a total of eight fish (*n* = 8; four fish per tank) were randomly selected for samples collection at the end of the experiment. Fish were euthanized with an overdose (200 mg L^−1^) of tricaine methanesulfonate (MS222; Argent Chemical Laboratories, Redmond, WA, USA). The exterior surface of fish was washed with 70% ethanol, and then an abdominal incision at the ventral midline was carried out to aseptically remove the whole intestine from the abdominal cavity. The distal intestine segment was dissected and the digesta was collected in a 1,5 ml eppendorf tube. However, only six fish had intestinal digesta at the sampling time (*n* = 6; three fish per tank). The digesta samples were flash frozen in liquid nitrogen, and stored at −80 °C until DNA extraction. At the time of the dissection, the swim bladder was still inflated, and was aseptically removed from same fish using different sterilized instruments to avoid crossover contamination. The anterior region of the swim bladder was transversally dissected in two ring-like sections. The swim bladder dissected sections were approximately 1.5 cm^2^. The two sections were immediately fixed either in 2% glutaraldehyde (Merck, Damstadt, Germany) or formalin solution, neutral buffered 10% (Sigma-Aldrich, St. Louis, MO, USA) for scanning electron microscopy or histology analyses, respectively. The remaining swim bladder tissue was washed in 15 ml of PBS (1X) in a 50 ml falcon tube, and gently vortex for 5 min at room temperature until the solution became cloudy. The wash step was repeated twice. The swim bladder tissue was extracted from the tube, and the remained cloudy liquid was transferred into 1.5 ml tubes and centrifuged at 13.000 × g for 1 min. The supernatant was discarded and the obtained pellet was stored at −80 °C until DNA extraction.

### DNA extraction and sequencing

DNA was extracted from the swim bladder-derived pellet, distal intestinal digesta samples (0.25 g) and the tank water samples using the MO BIO PowerFecal^®^DNA Isolation Kit (MO BIO Laboratories, Carlsbad, CA, USA) according to the manufacturer’s protocol. DNA concentrations were determined using the Qubit® dsDNA HS Assay kit (Life Technologies, Grand Island, NY, USA). The V4 region of the 16S rRNA gene was amplified following the fusion primer method using the primers 515F (GTGCCAGCMGCCGCGGTAA) and 806R (GGACTACHVGGGTWTCTAAT). Variable region 4 was selected because of its high coverage, low error rate and minimum loss of taxonomic resolution^11,12^. The resulting amplicons were of suitable length to be used in the Ion Torrent^TM^ sequencing platform (Life Technologies). All PCR reactions were performed in duplicates in a 30 µL reaction mixture containing 1.5 U (5U/µL) GoTaq® G2 Flexi DNA Polymerase (Promega, WI, USA), 6 µL of Buffer (5X), 2.4 µL of Mg (25 mM), 1.2 µL of nucleotides mix (5 mM each), 0.3 µL of primers (20 µM) and 18.5 µL of nuclease free water. In addition, a negative PCR control without the DNA template was run. The PCR conditions included an initial denaturation at 94 °C for 5 min, followed by 35 cycles of denaturation at 94 °C for 30 sec, annealing at 56 °C for 30 sec, and extension at 68 °C for 45 sec. After the cycling procedure, the amplicons from each sample were pooled and run on a 1% agarose gel. Subsequently, the amplicons were purified with the QIAquick® PCR Purification kit (Qiagen). DNA sequencing was performed via the Ion Torrent at the facilities of the University of Plymouth Enterprise Ltd. An Ion Library Quantitation Kit (Life Technologies, CA, USA) was used to evaluate amplicons for fragment concentration. Library concentration was adjusted to 26 pM for all samples. For template preparation, amplicon fragments were attached to Ion Sphere Particles using an Ion PGM Template OT2 400 kit (Life technologies, USA), following manufacture’s protocol.

### Bioinformatics analyses

Sequencing reads of the 16 S rRNA gene were processed and analyzed using UPARSE^13^ and QIIME^14^. The quality of the reads was assessed using FastQC Software. The reads were truncated at 270 bp and filtered for quality using a maximum expected error value of 0.01. Filtered reads were dereplicated and singletons were removed. These sequences were clustered into OTUs at a 97% similarity cutoff following UPARSE pipeline. OTUs were classified using a Naive Bayes classifier and the Ribosomal Database Project (RDP) taxonomic database, with probability cutoff of 97%. Chloroplast and Archaea sequences as well as sequences that were unclassified at the kingdom level were removed. Sequences that had a frequency lower than 10 were removed from the dataset. To avoid biases generated by differences in sequencing depth, each sample was subsampled to an even depth of 52,000 sequences. The analyses of diversity indexes, including Good’s coverage, alpha diversity indexes comprising community diversity (Simpson and Shannon index), richness (Chao-1) and phylogeny-based metrics (PD Whole Tree), were determined using QIIME. Principal coordinates analysis (PCoA) was conducted to evaluate the Beta diversity obtained by unweighted UniFrac and weighted UniFrac analyses, using the “beta_diversity.py” QIIME script. EMPeror was used to represent the PCoA plots from the unweighted and weighted UniFrac metrics. The predicted functional analysis were performed using PICRUSt as described by Ramirez and Romero^15^. The accuracy of the predictions of the functional potential of the bacteria communities based on the 16 S rDNA sequences was assessed by computing the Nearest Sequenced Taxon Index (NSTI), which shows the relationship of the microbes under evaluation to the bacterial genomes in a database. The associated metabolic pathways were deciphered by employing HUMAnN2 (The HMP Unified Metabolic Analysis Network) with the default settings.

### Scanning electron microscopy analysis

Samples of the swim bladder were processed following Rojas *et al*.^16^. Briefly, samples were fixed with 2% glutataraldehyde (Merck, Damstadt, Germany), dehydrated in increasing concentrations of ethanol in 100%, and follow a drying step using liquid CO_2_ as transitional fluid. Samples were gold coated and examined on a JSM-T300 (JEOL) scanning electron microscope (Supplementary material).

### Histology

Swim bladder samples were fixed with formalin solution, neutral buffered, 10% (Sigma-Aldrich, St. Louis, MO, USA) during 48 hrs. Samples were dehydrated through graded alcohol series (70–100%), cleared in xylene and embedded in paraffin. Paraffin sections of 5 µm of thicknesses were cut and stained with hematoxylin–eosin, Alcian blue, Van Giesson and Red Hot Sir, and analyzed under an Olympus CX21 light microscope (Olympus, Center Valley, PA, USA) (Supplementary material).

### Statistical analyses

Shannon Diversity index, Simpson index, richness and PD Whole Tree were analyzed to determine the differences in alpha diversity between swim bladder microbiota and distal intestinal digesta microbiota. Normality was tested with Shapiro and Wilk test. Comparisons were performed either using student t-test or Mann-Whitney test for normal or non-normal data distribution, respectively. The analyses were run in GraphPad Prism 6 software (GraphPad Software, Inc., La Jolla, CA, USA) with a 5% of significance level. The UniFrac distance matrices were analyzed by Analysis of Similarities (ANOSIM) and Permutation multivariate analysis of variance (PERMANOVA) with 9999 permutations. For this purpose, the dissimilarity matrix for the unweighted and weighted UniFrac was analyzed in QIIME with a 5% of significance level. All OTUs with same taxonomic classification were agglomerated by looking for differential abundance of agglomerated counts at the phylum through genus levels. DESeq2 software package^17^ was used to determine differences in abundance of taxonomic levels (i.e., phylum or genus) between swim bladder and distal intestine digesta, considering a significance level of 5%. Additionally, we conducted a t-test analysis with *P* values corrected with the Benjamini–Hochberg false discovery rate method to identify bacterial functional pathways that showed differences in relative abundance between swim bladder microbiota and distal intestinal digesta.

### Ethics approval

The study was conducted in accordance with the guidelines of the Bioethics and Biosecurity committee of the Instituto de Nutrición y Tecnología de los Alimentos (INTA) at Universidad de Chile.

## Results

### Sequencing depth

A total of 1,529,645 high-quality sequences longer than 270 bp, with an average of 95,603 sequences/sample, were retained after quality filtering and processing sequencing reads. Dataset was representative of bacterial communities due to Good’s coverage estimators for all samples were greater than 99%. The sequencing depth was evaluated by observing the saturation phase of the rarefaction curves based on alpha diversity metrics (Chao1 and PD whole tree) at 30,000 sequence readings in both swim bladder and distal intestinal content samples.

### Diversity analysis of microbiota of swim bladder and distal intestinal digesta

**Figure 1:**
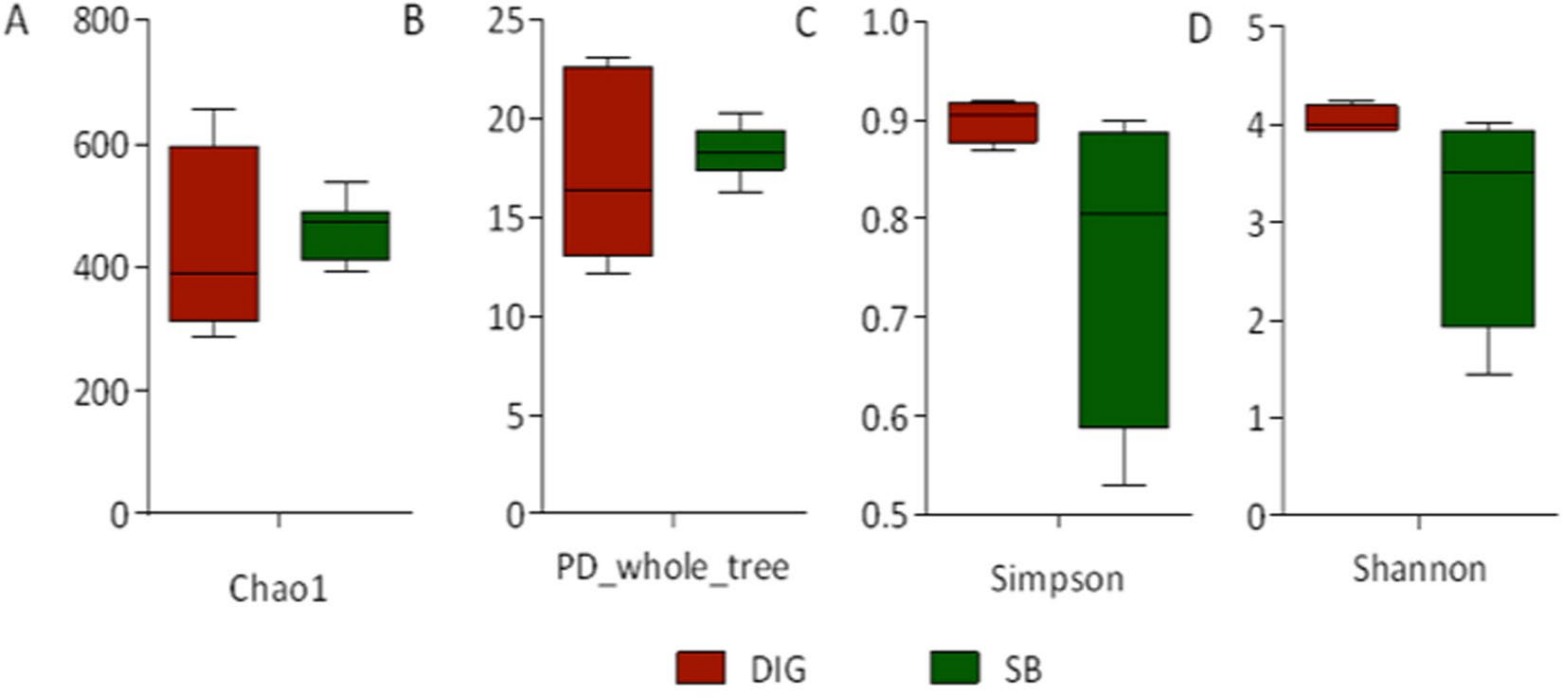
Comparison of alpha diversity indexes between the swim bladder resident microbiota (SB) and the distal gut digesta microbiota (DIG) in rainbow trout (*Oncorhynchus mykiss*). Diversity in the swim bladder bacteria and the distal gut digesta bacteria community was measured using Chao-1 (**A**), phylogeny-based metrics (**B**), simpson index (**C**) and shannon index (**D**). A student-test was used to determine significance differences in Chao-1, phylogeny-based metrics and Shannon index using a critical value of α = 0.05. A Mann-Whitney test was used to determine significance differences in Simpson index using a critical value of α = 0.05. No significant differences were detected for any alpha diversity indexes.

**Figure 2:**
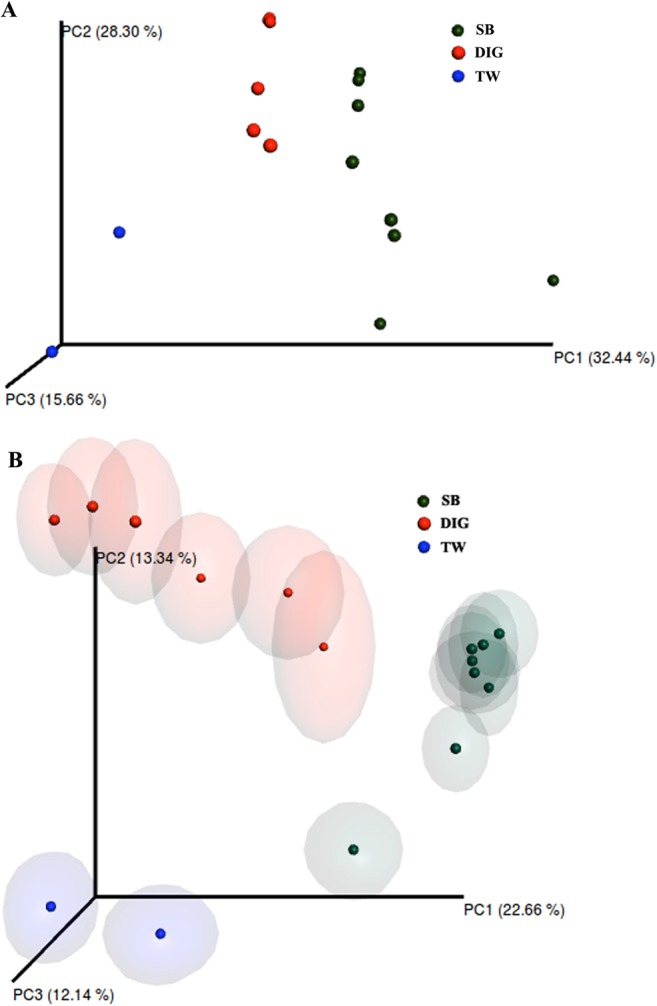
Principal coordinates analysis (PCoA) of the bacterial communities derived from the weighted (**A**) and unweighted (**B**) UniFrac distance matrix. Circles represent individual samples from swim bladder microbiota (green circles; *n* = 8) and distal gut digesta microbiota (red circles; *n* = 6) from *Oncorhynchus mykiss*. Blue circles (*n* = 2) represent microbiota from water samples of experimental tanks; these samples were not included in the beta diversity analysis. The confidence ellipsoids around sample symbols display the degree of variation from one sample to the next.

We detected no significant differences in the alpha diversity indexes when comparing swim bladder-associated microbiota with distal intestinal digesta microbiota (Fig. 1). Further, we conducted a beta diversity analysis to determine either similarity or dissimilarity in the composition of the microbiota found in the swim bladder tissue, distal intestinal digesta and tank water. These results were depicted in the principal coordinates plot using the weighted and unweighted UniFrac distances (Fig. 2A,B, respectively). In the weighted UniFrac analysis, the two main components explain 60.7% of the observed variance (first component, 32.4% and second component, 28.3%). On the other hand, in the unweighted UniFrac analysis, the two main components explain 36% of the observed variance (first component, 22.6% and the second component, 13.3%). This means greater variance was observed when considering the taxa relative abundance data. The water tank bacterial communities samples were added exclusively to illustrate how far they were located in the space with regard swim bladder and distal intestine digesta. However, water tank samples were not included in the statistical analysis. The PERMANOVA and ANOSIM tests showed significant differences (*P* < 0.001) in the composition of the microbiota between swim bladder or distal intestine digesta, in both the weighted and the unweight UniFrac analyses (Table 1).

**Table 1:** PERMANOVA and ANOSIM test results for comparisons of microbiota composition between swim bladder and distal intestine digesta samples in rainbow trout.

	Statistical Test	Test Statistic	*P* value
Unweighted UniFrac	PERMANOVA	3.067	0.0001**
	ANOSIM	0.798	0.0001**
Weighted UniFrac	PERMANOVA	5.343	0.0001**
	ANOSIM	0.489	0.0004**

**Indicates a rejection of the null hypothesis of no differences among groups (*P* < 0.001).

### Differences in relative abundance of microbial taxonomic composition of swim bladder and distal intestine digesta

**Figure 3:**
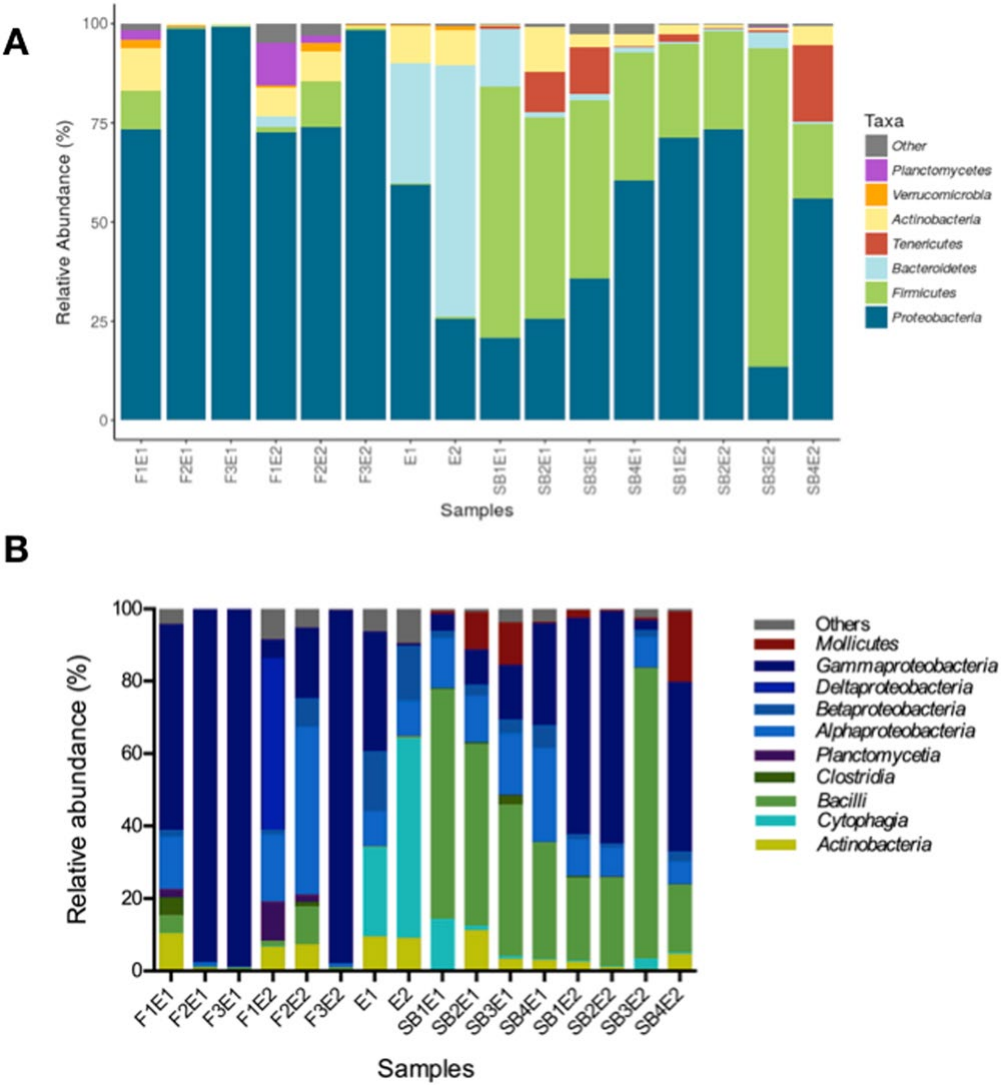
Relative abundance (%) at the phylum (**A**) and Class level (**B**) of each sample obtained from the microbiota of the swim bladder and the microbiota of the distal intestine digesta in rainbow trout, and from the water samples microbiota obtained from experimental tanks. Swim bladder samples from tank 1; SB1E1, SB2E1, SB3E1 and SB4E1, and from tank 2; SB1E2, SB2E2, SB3E2 and SB4E2. Distal intestine digesta samples from tank 1; F1E1, F2E1 and F3E1, and from tank 2; F1E2, F2E2 and F3E2. Water sample from tank 1; E1, and from tank 2; E2.

In the swim bladder, predominant phyla were *Proteobacteria* and *Firmicutes*, while *Proteobacteria* was the most abundant phylum in distal intestinal digesta (Fig. 3A). Further, in tank water samples, the most abundant phyla were *Bacteroidetes* and *Proteobacteria*. At the class taxonomic level, *Bacilli* and *Gammaproteobacteria* were the dominant classes in swim bladder, while *Gammaproteobacteria* was the dominant class in distal intestine digesta (Fig. 3B). Further, *Cytophagia* was the most abundant class in tank water samples.

**Figure 4:**
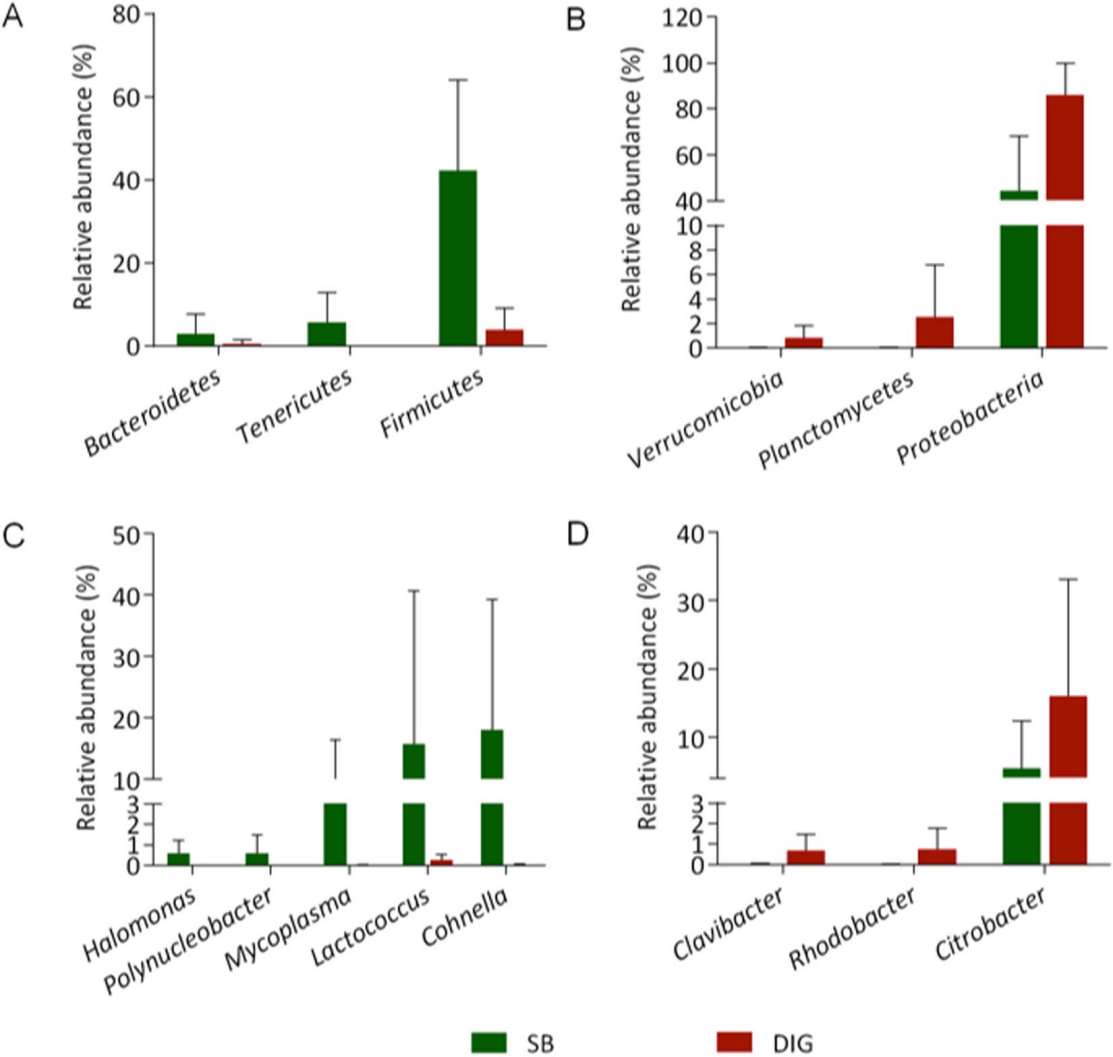
Comparison of bacterial communities between the microbiota of the swim bladder and the microbiota of the distal intestine digesta in rainbow trout. Statistical significant taxa are represented in accordance to DESeq2 analysis. Graphics illustrate the differences in the microbiota composition based on the relative abundance of taxa (phylum and genus). Section (**A**,**B**) depicts the most abundant phyla observed in the microbiota from the swim bladder and from distal intestine digesta, respectively in the rainbow trout. Section (**C**,**D**) highlights the most abundant bacterial genera observed in the microbiota from the swim bladder and from the distal intestine digesta, respectively in the rainbow trout.

Differential abundance at phylum and genus level between distal intestine digesta and swim bladder are represented in Table 2. In addition, the relative abundance of these components is depicted in Fig. 4. At the phylum level, *Firmicutes*, *Tenericutes and Bacteroidetes* showed greater relative abundance in the swim bladder microbiota compared to distal intestine digesta microbiota in rainbow trout (Fig. 4A). On the other hand, *Proteobacteria*, *Planctomycetes* and *Verrucomicobia* phyla were relatively more abundant in distal intestine digesta microbiota in fish (Fig. 4B). Bacterial genera, including *Cohnella*, *Lactococcus*, *Mycoplasma*, *Polynucleobacter* and *Halomonas* showed greater relative abundance in the swim bladder microbiota compared to distal intestine digesta microbiota in rainbow trout (Fig. 4C). *Clavibacter*, *Rhodobacter* and *Citrobacter* bacterial genera were relatively more abundant in the distal intestine digesta microbiota (Fig. 4D). Finally, *Acinetobacter* and *Sphingomonas* bacterial genera, although with lower significant magnitude than those observed for the genera above-mentioned, were significantly less abundant in the swim bladder microbiota compared to the distal intestine digesta microbiota in the rainbow trout (Table 2).

**Table 2:** Summary of differential abundance at phylum and genus level between distal intestine digesta and swim bladder in rainbow trout.

Phylum	Genus	Log 2 fold difference^a^	*P* adjusted^b^
Firmicutes		3.5035	<0.001
	Cohnella	4.2391	<0.001
	Lactococcus	2.1907	0.017
Bacteroidetes		1.8064	0.003
Tenericutes		6.386	<0.001
	Mycoplasma	4.0288	<0.001
**Actinobacteria**			
	Clavibacter	−8.0276	<0.001
Proteobacteria		−1.9962	0.004
	Rhodobacter	−8.3502	<0.001
	Citrobacter	−5.5939	<0.001
	Sphingomonas	−3.5561	<0.001
	Acinetobacter	−2.491	<0.001
	Halomonas	2.7042	<0.001
	Polynucleobacter	2.3841	0.002
Planctomycetes		−4.0278	<0.001
Verrucomicrobia		−4.6094	<0.001

^a^Analysis done using DESeq2 package. Positive values indicate higher abundance in swim bladder compared to distal intestine digesta. Negative values indicate lower abundance in swim bladder compared to distal intestine digesta.

^b^Adjusted *P* value; accounts for multiple testing and controls the false discovery rate.

### Core microbiome for swim bladder and distal intestine digesta

**Figure 5:**
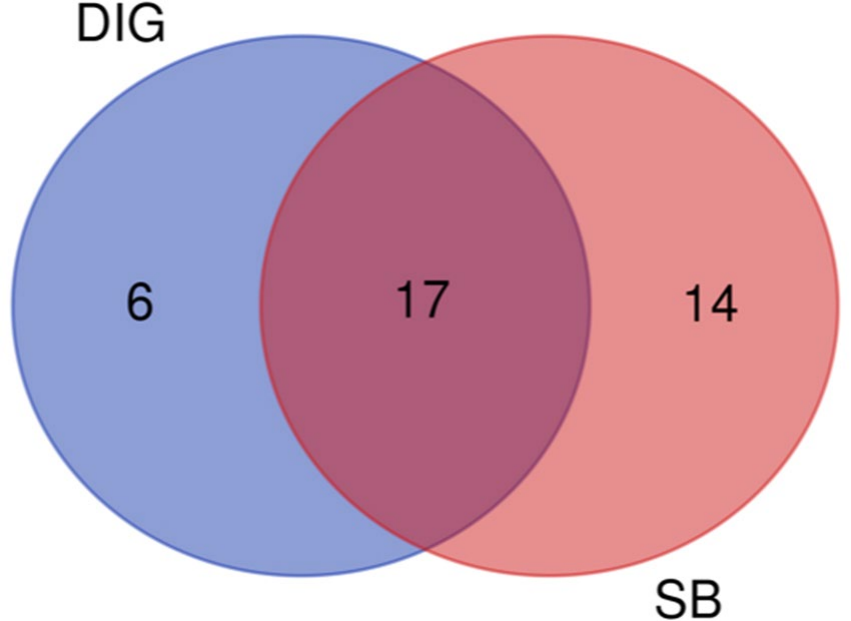
Venn diagram representing common genera between swim bladder (SB) and distal intestine digesta (DIG). Diagram shows numbers of genera present in at least 75% of all samples from each origin.

A core microbiome of 17 common genera, including Rhodobacter, Vagococcus, Leuconostoc, Clavibacter, Facklamia, Methylobacterium, Pseudomonas, Lactococcus, Flectobacillus, Corynebacterium, Staphylococcus, Aerococcus, Acinetobacter, Cohnella, Enhydrobacter, Lactobacillus and Citrobacter, were detected for swim bladder and distal intestine digesta (present at least in 75% of samples in each compartment) (Fig. 5). Six genera, including Planctomyces, Jeotgalicoccus, Dietzia, Mycobacterium, Legionella and Luteolibacter, were exclusive for distal intestine digesta. Finally, 14 genera, including Streptococcus, Novosphingobium, Sphingomonas, Clostridium, Photobacterium, Halomonas, Mycoplasma, Ralstonia, Desemzia, Bradyrhizobium, Sediminibacterium, Bacillus, Polynucleobacter and Yersinia, were exclusive for swim bladder.

### Prediction of metabolic functions of bacterial communities

**Figure 6:**
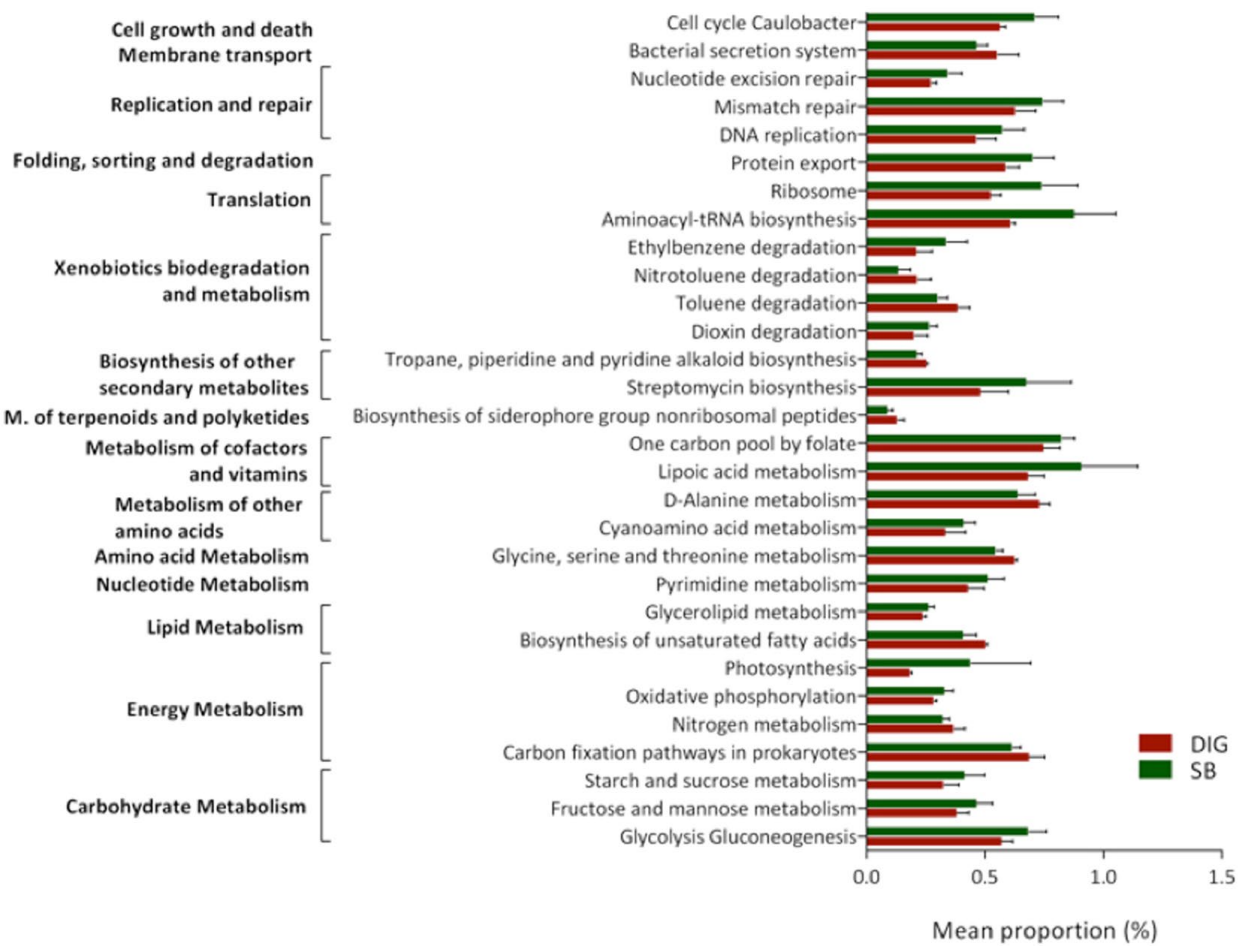
Comparison of the presumptive metabolic function analysis (PICRUSt) between the microbiota from the swim bladder (SB) and the microbiota from the distal intestine digesta (DIG) in rainbow trout. All represented pathways were significantly (*P* < 0.05) different. Differences were detected using a *t*-test with a critical value of *α* = 0.05. *P*-values were corrected with the Benjamini-Hochberg false discovery rate method.

The PICRUSt analysis predicts metabolic functions associated with bacteria community based on 16 S rRNA data^18^. We evaluated whether differences exist at the metabolic function level between swim bladder microbiota and distal intestine digesta microbiota. The accuracy of the prediction was determined by computing the Nearest Sequenced Taxon Index (NSTI), revealing a mean of 0.13 ± 0.04 for distal intestine digesta and 0.13 ± 0.06 for swim bladder samples; this, indicates a relatively good match (87%) to reference genomes (ideal NSTI ≤ 0.03^15^). Significant differences were assessed using t-test, and P-values were corrected with the Benjamini–Hochberg false discovery rate method (FDR). A total of 30 metabolic pathways, clustered in 15 major metabolic functions, were found to be different (*P* < 0.05) between the identified bacterial communities of the swim bladder and the bacterial communities of distal intestine digesta (Fig. 6). PICRUSt indicates 20 metabolic pathways to be more abundant (*P* < 0.05) in the swim bladder microbiota compared to the distal intestine digesta microbiota. These pathways included carbohydrate metabolism, oxidative phosphorylation, nucleotide metabolism, lipoic acid metabolism, translation, folding, sorting and degradation, replication and repair. On the other hand, metabolic pathways including, nitrogen metabolism, biosynthesis of unsaturated fatty acids, amino acid metabolism, degradation of both toluene and nitrotoluene as well as bacterial secretion system, were found to be greatly abundant (*P* < 0.05) in the microbiota of distal intestine digesta compared to microbiota of the swim bladder. The significant differences in the predicted bacterial functions are detailed in Table 3 (supplementary material).

### Histology and scanning electron microscopy

We observed sections with simple cuboidal epithelium with great amount of mucine secretion on the surface, sections with soft folds with pseudostratified columnar epithelium and sections with extremely thin and pronounced folds in the inner wall mucosa of the swim bladder (Supplementary material: Image A, B and C, respectively). Overall, each mucosa section is coated by an organized epithelium with highly mucine secretory cells (positive alcian blue stain) and patches of ciliated cells (Supplementary material: image D). Further, a dense irregular connective tissue with collagen type I (highly acidophilic) underlies the mucosa epithelium (Supplementary material: image A). The connective tissue goes through all the swim bladder mucosa, and as part of the different fold structures described previously. In addition, beneath the connective tissue there is smooth muscle tissue, which arises as the main structural component of the swim bladder wall (Supplementary material: image E and F). The smooth muscle is organized as inner circular layer and external longitudinal layer (Supplementary material: image E). It is possible to observe nerve fibers, neuronal corpuscles and blood vessels between both smooth muscle layers. Scanning Electron Microscopy revealed patches of ciliated cell cumulus in the swim bladder mucosa of rainbow trout, similar to those observed in the low airways mucosa of mammalian lung, and profuse mucus-like secretion in the surface of the mucosa of the swim bladder.

## Discussion

External and internal mucosal surfaces of metazoan organisms offer great diversity of ecological niches to microorganism that live in close association with host. In aquatic environments, microorganisms are abundant in both water and sediments^19^; aquatic microbial loads are greater than air and soil, therefore, fish are expose to greater microbes loads compared with terrestrial domesticated animals^20^. In fish, similar to other vertebrates, the major proportions of microbes reside in the gastrointestinal tract. Hence, most of microbiota research has focused in gut microbiota to elucidate its role in the physiology and health of fish. Less attention has been addressed to study in-depth the microbiota associated with anatomic compartments in contact with the environment other than the gastro-enteric tract, such as the swim bladder, in physostomous fish^9^.

In our study, beta diversity analyses revealed significant differences between microbiota associated with the swim bladder and microbiota of the distal intestine digesta of fish. Whilst *Firmicutes* was the most abundant phylum in the swim bladder-associated microbiota, *Proteobacteria* was the dominant phylum in distal intestine digesta. Previous works have reported *Proteobacteria* and *Firmicutes* as majors phyla observed in gut content of aquaculture fish^15,21–27^. However, the relative abundance of these phyla, varies between studies. This is especially true, since intestinal microbiota is highly modulated by a plethora of factors, including rearing environmental conditions (i.e., seawater *versus* freshwater; aquaculture *versus* wild; cold water *versus* warm water), ontogenic phase, tropich level, gastroenteric segment and diet composition^8^. In the case of diet, protein source modulates the microbial communities in distal intestine of fish, particularly in carnivorous fish (i.e., salmonids) fed plant-derived protein^8,22,24,28^ Here, we fed a high soy protein concentrate (38%) diet to rainbow trout. In agreement with Desai *et al*. (2012)^22^, we observed *Proteobacteria* to be dominant phyla (>60%) in distal intestinal contents of rainbow trout fed a high soy protein concentrate diet (30%). Here, at the genus level, *Cohnella*, *Lactococcus and Mycoplasma* were more abundant in the swim bladder-associated microbiota, *Citrobacter*, *Rhodobacter* and *Clavibacter* were more abundant in the distal intestine digesta microbiota. We suggest this might obey differences in ecological niches determined by physicochemical characteristics of the anatomic compartment; oxygen tension levels, pH values and nutrient availability, might contribute with selection pressure on bacterial species in favor of those able to survive in the conditions prevailing at the anatomic compartment. It has been argue the existence of a hyperoxic environment prevailing in the swim bladder due to high oxygen partial pressures^29,30^, and thus constituting a physicochemical barrier that might restrict bacterial growth in favor of those capable to deal with high oxygen tension. Interestingly, the detection of a core microbiota, represented by 17 genera, common for swim bladder and distal intestine digesta, suggests that these bacteria are well-adapted to survive in different anatomical compartment in fish. However, whether this core microbiota is common in the swim bladder of all physostomous species, regardless their trophic level, requires further study.

The presumptive functional analysis revealed greater abundance of oxidative phosphorylation pathway in bacterial communities of the swim bladder compared to distal intestinal digesta microbiota. This evidence suggests aerobic metabolism might be use to oxidize energy-yielding substrates in swim bladder microbiota. Moreover, lipoic acid metabolism was found to be significantly more abundant in swim bladder bacteria than distal intestinal content bacteria. Lipoic acid is an essential enzyme cofactor universally required for aerobic metabolism^31^. Therefore, greater lipoic acid metabolism suggests higher aerobic metabolism via oxygen consumption in swim bladder bacteria compared with distal intestinal content bacteria. Additionally, the swim bladder hyperoxic environment might exert a chemical barrier for bacteria growth via generation of oxygen-derived free radicals (ROSs). It is well known organism expose to increasing oxygen concentrations promotes greater production of ROSs (i.e., superoxide and H_2_O_2_)^32,33^. The increased levels of ROSs might impair microorganism growth via oxidative damage in several cellular components, such as proteins and DNA^34^. However, bacteria have developed defensive mechanism, including DNA repair systems, in order to repair oxidized DNA^35,36^. Here, we detected greater abundance of pathways related with cellular growth and cellular death, translation, nucleotide excision and mismatches repair as well as DNA replication in the swim bladder-associated bacteria compared to distal intestinal digesta bacteria. Thus, it appears greater abundance of both DNA repair systems and DNA replication pathways constitute bacterial adaptive mechanisms to deal with the prevailing pro-oxidative condition of the hyperoxic environment in the swim bladder.

Nutrient availability and substrate affinity constitute other factors that contribute with bacterial diversity. The swim bladder-associated bacteria greatly depend on epithelia mucus secretion (i.e. mucin, a heavily glycosylated protein) and cellular detritus to cover their nutrients and energy requirements for maintenance and growth. In our study, the histological analysis revealed great amount of mucin secretory cells (positive alcian blue stain) and great amount of mucin secretion in sections of the swim bladder epithelium, including the surface of ciliated cells (Supplemented material; image A, D and F). Further, the predictive function analysis revealed greater abundance of carbohydrates metabolism pathway, including starch, sucrose, mannose and fructose metabolism as well as glycolysis and gluconeogenesis pathway, in bacterial communities of the swim bladder. These findings suggest carbohydrates, such as those of mucin secretion, might constitute an energy-yielding source to swim bladder-associated bacteria. On the other hand, gut microbiota are able to metabolize ingested feed-derived nutrients, such as amino acids and lipids^37^. Salmonids diets include great amount of protein and lipids as major energy-yielding nutrients. We detected greater abundance of amino acid metabolism, including glycine, serine, threonine and D-alanine metabolism in bacterial communities of the distal intestine digesta compare with swim bladder-associated bacteria; this, most likely due to intestinal bacterial utilize dietary proteins for maintenance and growth. Further, we found lower abundance of unsaturated fatty acid biosynthesis pathway in swim bladder-associated bacteria compared with distal intestine digesta bacteria. It is well known unsaturated fatty acids are susceptible to oxidative damage by ROS^38^. Due to prevailing pro-oxidant conditions derived from hyperoxic environment of the swim bladder^5,39^, we suggest swim bladder-associated bacteria modulate their metabolism to cope with an hyperoxic environment, reducing unsaturated fatty acid biosynthesis to minimize the production of substrate prone to lipid-peroxidation.

The bacterial route of access and factors underlying the successful colonization of ecological niches of the swim bladder in physostomous has not been studied yet. Although, water and feed constitute major factors modulating fish gut microbiota^40–42^, it appears water microbiota does not exert significant influence in swim bladder bacteria composition since we found no similarities between the water bacterial communities and the swim bladder bacteria at the phylum level. Therefore, gulping air from water surface during the inflation of the swim bladder, might constitute a feasible mechanism for mobilizing bacteria from the air-water interface toward the swim bladder mucosa in physostomous; similarly to bacteria colonization of higher vertebrate respiratory system^43–46^. In the past, the structures of human respiratory system were thought to be sterile unless infected; however, recent studies have provided strong evidence revealing diverse communities of bacteria in both healthy and diseased lungs^43–46^. The study of bacterial dysbiosis in chronic lung disorders, including asthmatic lung, chronic obstructive lung diseases and cystic fibrosis lung, has gained increasing attention for a better understanding of the host–microorganism interactions in human^46^. The above-mentioned respiratory disorders are characterized, among other aspects, by an increase in the secretion of mucin and the relative abundance of *Proteobacteria* and *Firmicutes* bacteria^46–48^. Interestingly, these are two observations we detected in the physostomous swim bladder of rainbow trout in our study. The homology of the lung and swim bladder has been well described based on morphological and embryological evidence in the past^1^. Zheng *et al*.^1^ found molecular homology at the transcriptome level between the physostomous zebrafish swim bladder and mammalian lung (i.e., human and mouse). Surfactant, a mixture of various lipids and surfactant proteins, is produced in both the swim bladder and the lung. However, the surfactant in the swim bladder has greater amount of cholesterol and unsaturated dipalmitoylphospholipids than in mammalian lung surfactant, most likely due to an antiglue-like activity to avoid permanent collapse of the bladder^49^.

Similarly to mammalian lung, we observed ciliated cells in the swim bladder of the rainbow trout (Supplementary material; image F, H and I). Brooks (1970)^50^ reported ciliated cells in rainbow trout swim bladder. Although the swim bladder in this species does not function as a respiratory organ, the author suggested the ciliated cells most likely facilitate moving mucous-like material secreted from the lamellar bodies to the pneumatic duct.

Taking in consideration the above-mentioned common aspects between the physostomous swim bladder and the mammalian lung, further research to explore the potential use of the physostomous swim bladder microbiome, as complementary biological model to study the role of microbiota in lung diseases in human, is required. This is not a mere speculation since the physostomous swim bladder of the zebrafish has already been used as a biological model to investigate, for example, human mucosal candidiasis^51,52^. Finally, the presence of microbial communities associated with the swim bladder suggests a potential role of this microbial ecology in the function of this organ, for example, contributing with swim bladder inflation process. In larvae, initial swim bladder inflation takes place during a critical window period in early development, and failing in this process implies expending more energy maintaining position in the water column due to negative buoyancy, thus diminishing juvenile growth performance^2^. In the past years, abiotic and biotic factors have been reported to influence swim bladder inflation process; however, there are still gaps that require further exploration in this field^2^. In this regard, improving our knowledge of swim bladder microbiota ecology will contribute with a better understanding of its role in swim bladder inflation, and thus with the well-being of aquaculture industry, particularly marine aquaculture. This is especially true, since reduced rates of swim bladder inflation during the hatchery phase of intensive larviculture significantly impact on the commercial production of marine fish species. Therefore, achieving relevant improvements in swim bladder inflation rate remains a top priority for the larval rearing industry in marine aquaculture^2^.

## Conclusion

This is the first in-depth characterization of the bacterial microorganism associated with the swim bladder in a physostomous species, such as the rainbow trout. Differences in the composition of bacterial communities at the phylum and genus level as well as in the predicted metabolic function analysis between swim bladder mucosa-associated bacteria and distal intestine digesta bacteria were detected. These differences might be consequence of different selection pressure occurring in these environments, selecting for different microbial community composition. Finally, increasing our knowledge of swim bladder microbiota will help to elucidate the role of this microbial ecology in swim bladder health and functionality, and thus to better understand its potential role in swim bladder inflation process. This is of great relevance in commercial production of marine fish species, since the failure of swim bladder inflation remains a major problem in intensive larval culture in commercial hatcheries.

## Supplementary information

Supplementary info

## Data Availability

Sequence data have been deposited in the Sequence Read Archive (SRA) of the National Centre for Biotechnology Information (NCBI) under the SRA accession: SRP144488.
