# Supplementary material for: In-depth analysis of swim bladder-associated microbiota in rainbow trout (Oncorhynchus mykiss)
Source: Sci Rep. 2019 Jun 20;9:8974. doi: 10.1038/s41598-019-45451-1 (PMC6586864; doi:10.1038/s41598-019-45451-1)
Supplement: Supplementary file 1 — Supplementary info [file 41598_2019_45451_MOESM1_ESM.docx]

**In-depth analysis of swim bladder-associated microbiota in rainbow trout (*Oncorhynchus mykiss*)**

Alejandro Villasante^a^; Carolina Ramírez^a^; Héctor Rodríguez^b^, Natalia Catalán^a^, Osmán Díaz^a^, Rodrigo Rojas^c^, Rafael Opazo^a^, Jaime Romero^a*^.

^a^ Laboratorio de Biotecnología de Alimentos, Unidad de Alimentos, Instituto de Nutrición y Tecnología de los Alimentos (INTA), Universidad de Chile, Santiago, Chile.

^b^ Facultad de Medicina, Universidad de Chile, Programa de Anatomía y Biología del Desarrollo, Santiago, Chile.

^c^ Laboratorio de Patobiología Acuática, Departamento de Acuicultura, Facultad de Ciencias del Mar, Universidad Católica del Norte.

^*^ [jromero@inta.uchile.cl](mailto:jromero@inta.uchile.cl)

^*^+56 2 2978 1524

**Connective**

**tissue**

**A**

**B**

**C**

**Mucine secretion**

**Ciliated cells**

**D**

The inner mucosa of the swim bladder exhibits some transition in morphology based upon the site under evaluation and the functional state of the organ. Some sites of the mucosa show a simple cuboidal epithelium with great amount of mucine secretion on the surface (A), sites with soft folds and a pseudostratified columnar epithelium (B) and sites with extremely thin and pronounced folds (C). The sites are coated by an organized epithelium with highly mucine secretory cells (positive alcian blue stain) and patches of ciliated cells (D). Further, a dense irregular connective tissue with collagen type I (acidophilic) underlies the mucosa epithelium (A).

**Inner circular layer**

**E**

**External longitudinal layer**

**Inner circular layer**

**External longitudinal layer**

**F**

Beneath the connective tissue there is smooth muscle tissue, which arises as the main structural component of the swim bladder wall (Supplementary material: image E and F). The smooth muscle is organized as inner circular layer and external longitudinal layer. It is possible to observe nerve fibers, neuronal corpuscles and blood vessels between both smooth muscle layers.

**F**

**G**

**A**

Image F) and G) highlight similarity of cellular structures of the swim bladder mucosa of physostomous fish and the low airways mucosa in mammalian lung. F) Scanning electron microscopy image revealing patches of ciliated cell cumulus in the swim bladder mucosa of rainbow trout (*Oncorhynchus mykiss*, this study). G) Scanning electron microscopy image of mono-stratified cuboidal epithelium of low airways, highlighting patches of ciliated cell cumulus in mammalian lung. Kindly provided by Charles Daghlian.

Direct access to G: http://remf.dartmouth.edu/images/mammalianLungSEM/image/lungtrachea1a.jpg

Web site

http://remf.dartmouth.edu/imagesindex.html

**I**

**H**

Scanning electron microscopy images (H and I) showing a profuse mucus-like secretion in the surface of the mucosa of the swim bladder of rainbow trout (*Oncorhynchus mykiss*, this study).

Table 3. Summary of differences in metabolic pathways between predicted metagenomes of the swim bladder (*n* = 8) and the distal intestine digesta (*n* = 6) in rainbow trout ^a^

| **KEGG pathways** | **Distal intestine digesta** | **Swim Bladder** | **FDR** |
| --- | --- | --- | --- |
| **Cell growth and death** |  |  |  |
| Cell cycle Caulobacter | 0.56 ± 0.02 | 0.71 ± 0.1**^*^** | 0.0047 |
| **Membrane transport** |  |  |  |
| Bacterial secretion system | 0.55 ± 0.09**^*^** | 0.46 ± 0.04 | 0.0394 |
| **Replication and repair** |  |  |  |
| Nucleotide excision repair | 0.27 ± 0.02 | 0.34 ± 0.06**^*^** | 0.0198 |
| Mismatch repair | 0.62 ± 0.08 | 0.74 ± 0.08**^*^** | 0.0295 |
| DNA replication | 0.46 ± 0.08 | 0.57 ± 0.09**^*^** | 0.0422 |
| **Folding, sorting and degradation** |  |  |  |
| Protein export | 0.58 ± 0.05 | 0.70 ± 0.08**^*^** | 0.0170 |
| **Translation** |  |  |  |
| Ribosome | 0.54 ± 0.04 | 0.73 ± 0.15**^*^** | 0.0058 |
| Aminoacyl-tRNA biosynthesis | 0.60 ± 0.01 | 0.87 ± 0.17**^*^** | 0.0030 |
| **Xenobiotics biodegradation and metabolism** |  |  |  |
| Ethylbenzene degradation | 0.20 ± 0.06 | 0.33 ± 0.08**^*^** | 0.0131 |
| Nitrotoluene degradation | 0.21 ± 0.05**^*^** | 0.13 ± 0.04 | 0.0164 |
| Toluene degradation | 0.38 ± 0.05**^*^** | 0.30 ± 0.04 | 0.0005 |
| Dioxin degradation | 0.20 ± 0.05 | 0.26 ± 0.03**^*^** | 0.0169 |
| **Biosynthesis of other secondary metabolites** |  |  |  |
| Tropane, piperidine and pyridine alkaloid biosynthesis | 0.25 ± 0.00**^*^** | 0.21 ± 0.02 | 0.0003 |
| Streptomycin biosynthesis | 0.48 ± 0.11 | 0.67 ± 0.19**^*^** | 0.0486 |
| **Metabolism of terpenoids and polyketides** |  |  |  |
| Biosynthesis of siderophore group nonribosomal peptides | 0.12 ± 0.02**^*^** | 0.08 ± 0.02 | 0.0134 |
| **Metabolism of cofactors and vitamins** |  |  |  |
| One carbon pool by folate | 0.74 ± 0.06 | 0.82 ± 0.05**^*^** | 0.0381 |
| Lipoic acid metabolism | 0.68 ± 0.06 | 0.90 ± 0.23**^*^** | 0.0430 |
| **Metabolism of other amino acids** |  |  |  |
| D-Alanine metabolism | 0.72 ± 0.04**^*^** | 0.63 ± 0.07 | 0.0215 |
| **Amino acid metabolism** |  |  |  |
| Glycine serine and threonine metabolism | 0.62 ± 0.01**^*^** | 0.54 ± 0.03 | 0.0000 |
| **Nucleotide metabolism** |  |  |  |
| Pyrimidine metabolism | 0.42 ± 0.06 | 0.51 ± 0.06**^*^** | 0.0394 |
| **Lipid metabolism** |  |  |  |
| Glycerolipid metabolism | 0.23 ± 0.01 | 0.26 ± 0.02**^*^** | 0.0320 |
| Biosynthesis of unsaturated fatty acids | 0.50 ± 0.08**^*^** | 0.40 ± 0.05 | 0.0014 |
| **Energy metabolism** |  |  |  |
| Oxidative phosphorylation | 0.28 ± 0.01 | 0.32 ± 0.03**^*^** | 0.0192 |
| Nitrogen metabolism | 0.36 ± 0.04**^*^** | 0.32 ± 0.02 | 0.0401 |
| **Carbohydrates metabolism** |  |  |  |
| Starch and sucrose metabolism | 0.32 ± 0.06 | 0.41 ± 0.08**^*^** | 0.0476 |
| Fructose and mannose metabolism | 0.38 ± 0.05 | 0.46 ± 0.06**^*^** | 0.0224 |
| Glycolysis and Gluconeogenesis | 0.57 ± 0.04 | 0.68 ± 0.07**^*^** | 0.0088 |

^a^ Mean values with their SD. Mean values marked with * were significantly (*P* < 0.05) greater regarding the condition to which was contrasted.

**1**

**2**

**J**
